# Use of Cerebrospinal Fluid for the Diagnosis of Neuroinvasive Dengue, Zika, and Chikungunya: A 19-year systematic review

**DOI:** 10.1590/0037-8682-0891-2020

**Published:** 2021-04-28

**Authors:** Cíntia da Silva Mello, Mauro Jorge Cabral-Castro, Luiz Claudio Silva de Faria, José Mauro Peralta, Marzia Puccioni-Sohler

**Affiliations:** 1 Universidade Federal do Rio de Janeiro, Programa de Pós-Graduação em Doenças Infecciosas e Parasitárias, Rio de Janeiro, RJ, Brasil.; 2 Universidade Federal do Estado do Rio de Janeiro, Escola de Medicina e Cirurgia, Rio de Janeiro, RJ, Brasil.; 3 Universidade Federal do Rio de Janeiro, Hospital Universitário Clementino Fraga Filho, Rio de Janeiro, RJ, Brasil.; 4 Universidade Federal do Rio de Janeiro, Instituto de Microbiologia Paulo de Góes, Rio de Janeiro, RJ, Brasil.

**Keywords:** Dengue virus, Zika virus, Chikungunya virus, Cerebrospinal fluid, Nervous system diseases

## Abstract

**INTRODUCTION:**

Cerebrospinal fluid analysis contributes to the diagnosis and neuropathogenesis of neuroinvasive arboviruses. Neurological complications caused by dengue, Zika, and chikungunya infections have high clinical relevance because of their high potential to cause death or neurological deficits. We aimed to evaluate the use of cerebrospinal fluid assays for diagnostic support in neurological disorders associated with dengue, chikungunya, and Zika infections.

**METHODS:**

A systematic review was carried out by searching the electronic databases LILACS, PubMed, Scopus, and Embase for articles written in English, Portuguese, or Spanish in the last 19 years. Published studies were reviewed using the terms “dengue,” “Zika”, “chikungunya”, alone or in combination with “cerebrospinal fluid” in the period from 2000 to 2019.

**RESULTS:**

A total of 98,060 studies were identified; of these, 1.1% (1,041 studies, 58,478 cases) used cerebrospinal fluid assays for neurological investigations. The most frequent neurological disorders included encephalitis (41.4%), congenital syndromes (17%), and microcephaly associated with Zika virus infections (8.9%). Neuroinvasive disorders were confirmed in 8.03% of 58,478 cases by specific cerebrospinal fluid analyses. The main methods used were IgM-specific antibodies (66%) and reverse transcription-polymerase chain reaction (10%). The largest number of scientific papers (29%) originated from Brazil, followed by India (18.4%) and the United States (14.4%).

**CONCLUSIONS:**

Although cerebrospinal fluid analysis is of great importance for increasing neurological diagnostic accuracy and contributes to the early diagnosis of neuroinvasive dengue, chikungunya, and Zika infections, it is underused in routine laboratory investigations worldwide.

## INTRODUCTION

Cerebrospinal fluid (CSF) is an ultrafiltrate of blood plasma produced by the choroid plexus cells. It protects the brain and spinal cord against trauma, provides nutrients to the nervous system tissue, and participates in the removal of residues generated by brain metabolism^1^ ^,^ ^2^. CSF examination dynamically reflects processes affecting the central nervous system (CNS). Analysis of the basic CSF composition (pleocytosis, mildly increased or normal protein, normal or mildly decreased glucose, and normal or mildly increased lactate levels) may contribute to the diagnosis of viral infections in the CNS^3^. Changes in these factors provide important information for the diagnosis of infectious and non-infectious neurological conditions^1^ ^,^ ^2^ *.* However, CSF findings may be similar for a variety of viral agents. Therefore, accurate diagnosis depends on clinical findings and special CSF laboratory tests such as specific molecular and immunological analyses.

Arboviruses are viruses that are transmitted to the vertebrate host through the bite of arthropod vectors, especially mosquitoes and ticks, and can be maintained in wild and/or urban cycles. Among the arboviruses, the main families that cause disease in humans are the *Flaviviridae, Togaviridae,* and *Bunyaviridae* families. Flaviviruses (dengue, Zika, and yellow fever viruses) and alphaviruses (chikungunya virus) are commonly transmitted by female mosquitoes of the genus *Aedes* (*Ae. aegypti* and *Ae. albopictus*) in a vertebrate human viremic host^4^. The broad distribution of vector mosquitoes associated with urbanization and climate change are some factors that facilitate the co-circulation of these agents, and consequently, the appearance of diseases caused by them^5^.

Scientific literature has reported an increase in the occurrence of neuroinvasive diseases after the simultaneous circulation of the dengue (DENV), Zika (ZIKV), and chikungunya (CHIKV) viruses. These include neurological disturbances of high morbidity and mortality such as acute encephalitis, aseptic meningitis, myelitis, and post-infection disorders such as Guillain-Barré syndrome, acute disseminated encephalomyelitis, optic neuromyelitis, cranial nerve palsy, and congenital disorders^6^ ^,^ ^7^ ^,^ ^8^ ^,^ ^9^ ^,^ ^10^ ^,^ ^11^ ^,^ ^12^ *.*

The diagnosis of neuroinvasive diseases associated with DENV, ZIKV, and CHIKV according to the Centers for Disease Control and Prevention (CDC) is based on the clinical criteria which include the appearance of neurological manifestations in the absence of a more likely clinical explanation, and are confirmed by laboratory criteria diagnosis, as follows: the presence of the following alone or in combination: viral RNA viral antigens in serum, CSF or other body fluids, specific IgM-reactivity in the CSF with non-reactive specific IgM reactivity for other arboviruses endemic in the region, virus-specific IgM in serum confirmed by virus-specific neutralizing antibodies, and increased titer-specific IgG in paired serum^13^ ^,^ ^14^ *.* Therefore, CSF examination has a role in demonstrating the etiological agent in viral infections of the CNS. The presence of viral genetic material in the CSF is considered to be the strongest evidence of causality^3^ ^,^ ^11^ ^,^ ^13^ ^,^ ^15^ ^,^ ^16^ ^,^ ^17^ *.*

The objective of this study was to conduct a review of the scientific literature on arboviruses (DENV, ZIKV, and CHIKV) that used the CSF assay for the investigation of neurological manifestations, over a period of 19 years. The results may elucidate the magnitude of CSF-assay use in arbovirus infections and demonstrate the importance of the topic; this would favor debate and interest in the scientific community about the importance of CSF assays for diagnostic support in neurological cases resulting from DENV, ZIKV, and CHIKV infections.

## METHODS

### Search Strategy

Published studies were reviewed using the terms “dengue”, “zika,” “chikungunya" alone and in combination with “cerebrospinal fluid” in the LILACS, PubMed, Scopus, and Embase databases. The guidelines used to report the systematic review were based on the Preferred Reporting Items for Systematic Reviews and Meta-Analysis (PRISMA) guidelines^18^. Articles written in English, Portuguese, or Spanish were selected in the period from January 1, 2000, to December 31, 2019. Articles were selected according to the presence of keywords in their titles and/or abstracts (based on the MeSH system). These documents were individually analyzed to remove duplicates in the databases. We also compared the numbers of total articles using the terms “dengue”, “Zika”, and “chikungunya” in the same 19-year period noted above. Two researchers independently searched for articles in different scientific databases and collected data, to avoid possible selection bias.

### Aspects Analyzed

The articles selected for this systematic review were reviewed in detail. We analyzed the total number of publications from 2000 to 2019, the number of publications per year and country, year of publication, and publication type (e.g., case reports, short communications, original articles, reviews, etc.). We also analyzed the number of confirmed neurologic diagnoses that involved infections by DENV, ZIKV, and CHIKV that used CSF as a screening sample. From these data, we developed graphs and tables in GraphPad Prism 8.0 and/or Microsoft Office Excel 2010. We analyzed the results obtained from the databases both separately and in combination. Duplicate articles were manually deleted using the duplicate removal function of the Mendeley desktop 1.19.4 program.

## RESULTS

### Selected Studies

**FIGURE 1:**
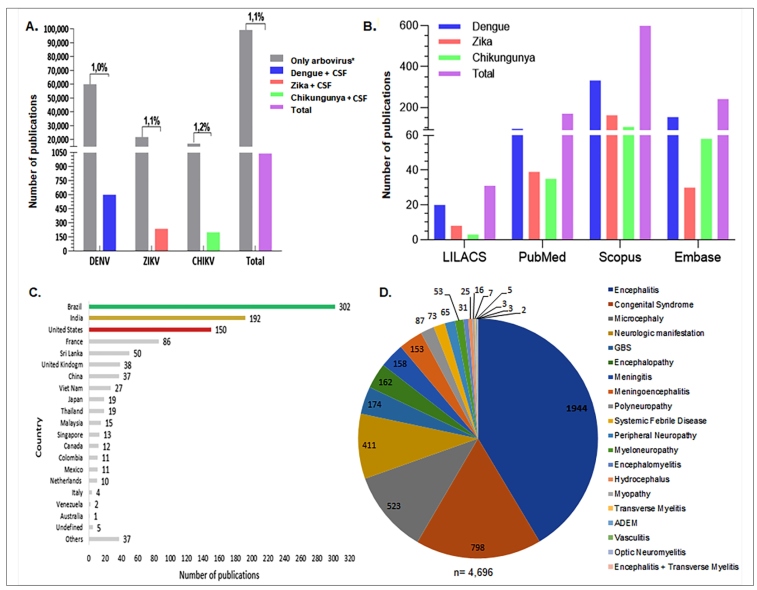
Number of publications in databases by country, and confirmed diagnoses in infections by arboviruses from 2000 to 2019, retrieved using the keywords “dengue”, “Zika”, “chikungunya”, and/or “CSF”. **(A)** The proportion of publications using CSF for the diagnostic evaluation of the total number over these 19 years filtered using the isolated words “dengue”, “Zika”, “chikungunya” in combination with “CSF” to the total number of publications using the arbovirus terms only. **(B)** Number of publications in the period in the LILACS, Pubmed, Scopus, and Embase databases. **(C)** Total number of publications by country retrieved from the databases. Descending order of the countries with the highest scientific production according to the body of authors and collaborators. **(D)** Number of confirmed diagnoses involving infections by DENV, ZIKV, and CHIKV that used the cerebrospinal fluid as a screening sample, originated from the publications filtered in the databases. GBS: *Guillain-Barré* Syndrome; ADEM: Acute Disseminated Encephalomyelitis. SIADH: Syndrome of Inappropriate Antidiuretic Hormone. *Only arbovirus = isolated “dengue”, “Zika” “chikungunya”.

The selection process of the articles for this study is presented in the flowchart presented in the Supplementary Figure. A total of 1,041 (1.1%) publications out of 98,060 articles were identified from the LILACS, PubMed, Scopus, and Embase databases using the keywords “dengue”, “Zika”, “chikungunya” alone and in combination with “cerebrospinal fluid”, from January 2000 to December 2019 (Figure 1A). The use of the term “dengue” with CSF resulted in the retrieval of 20 publications from LILACS, 96 from PubMed, 332 from Scopus, and 153 from Embase, totaling 601 publications. The use of the term “Zika” with CSF resulted in the retrieval of eight publications from LILACS, 39 from PubMed, 162 from Scopus, and 30 from Embase, totaling 239 publications. The use of the term “chikungunya” with CSF resulted in the retrieval of three publications from LILACS, 35 from PubMed, 105 from Scopus, and 58 from Embase, totaling 201 publications (Figure 1B). 

### Arbovirus-specific data

A total of 98,060 publications were found in the LILACS, PubMed, Scopus, and Embase databases using the single keywords “dengue,” “Zika”, and “chikungunya”. The proportion of publications using CSF for diagnostic evaluation over these 19 years was equivalent to only 1.1% (1,041) of the DENV, ZIKV, and CHIKV arbovirus articles (Figure 1A). For each specific arbovirus, the proportion of CSF-associated publications was as follows: DENV publications, 1.0% (601/59,503), ZIKV publications, 1.1% (239/21,575), and CHIKV publications, 1.2% (201/16,982) (Figure 1A).

### Principles Publication by Countries

According to the countries registered by the authors and collaborators, Brazil had the largest number of scientific papers available, accounting for 302 articles, from 2000 to 2019 (Figure 1C). This figure corresponds to 29.0% (302/1,041) of the publications. India was second with 192 publications (18.4% of 1,041), and the United States was third, with 150 publications (14.4% of 1,041) (Figure 1C).

### Neurological disorders and cerebrospinal fluid assay

In the 1,041 publications selected using the keywords “dengue”, “Zika”, and “chikungunya” both alone and in combination with “cerebrospinal fluid” over the 19 years, we identified 58,478 suspected cases of arbovirus and neurological disorders such as encephalitis, congenital syndromes, microcephaly, meningoencephalitis, meningitis, GBS, and myopathy. The CSF samples were investigated for arboviruses using different methodologies such as the detection of specific IgM and/or IgG antibodies, plate reduction neutralization test, detection of viral antigens, amplification of viral RNA by PCR techniques, and next-generation metagenomic sequencing. However, only 8.03% (4,696/58,478) were confirmed as DENV, ZIKV, and/or CHIKV cases by the analysis of CSF alone or in association with other fluids (Figure 1D). The diagnosis was confirmed in 66% (3,099/4,696) of these confirmed CSF cases by the detection of specific IgM, 10% (470/4,696) by RT-PCR, 0.15% (7/4,696) by viral isolation, 0.12% (6/4,696) based on the detection of the NS1 viral protein, and 23.7% (1,114/4,696) using other methods in CSF analysis. CSF alone was used for diagnosis in 75% (3,545/4,696) of the cases, CSF and serum in 22% (1,036/4,696) , CSF, serum, and urine in 0.26% (12/4,696), CSF and urine in 0.11% (5/4,696), serum alone in 1.81% (85/4,696), and other biological specimens (saliva, amniotic fluid, and autopsy) in 0.28% (13/4,696) of the cases. Encephalitis had the highest incidence, and was reported in 41.4% of the total confirmed cases (1,944/4,696) (Figure 1D). Congenital syndromes and microcephaly were reported in 17.0% (798/4,696) and 8.9% of cases (417/4,696) respectively, and occupied the second and third positions; both of the above conditions were related to ZIKV infections, and the diagnoses were confirmed by CSF analysis (Figure 1D). GBS ranked fifth in confirmed cases, accounting for 3.7% of the total cases (174/4,696) (Figure 1D).

### Publications overview

#### From 2000 to 2015

The number of publications that evaluated arbovirus-associated neurological manifestations in combination with CSF was low (n = 426; data not shown), considering all four databases. Except for those pertaining to DENV (341/426; data not shown), accessible works that related ZIKV (1/426; data not shown) and CHIKV (84/426; data not shown) to CSF during the reporting period were sparse.

#### From 2016 to 2019

**FIGURE 2:**
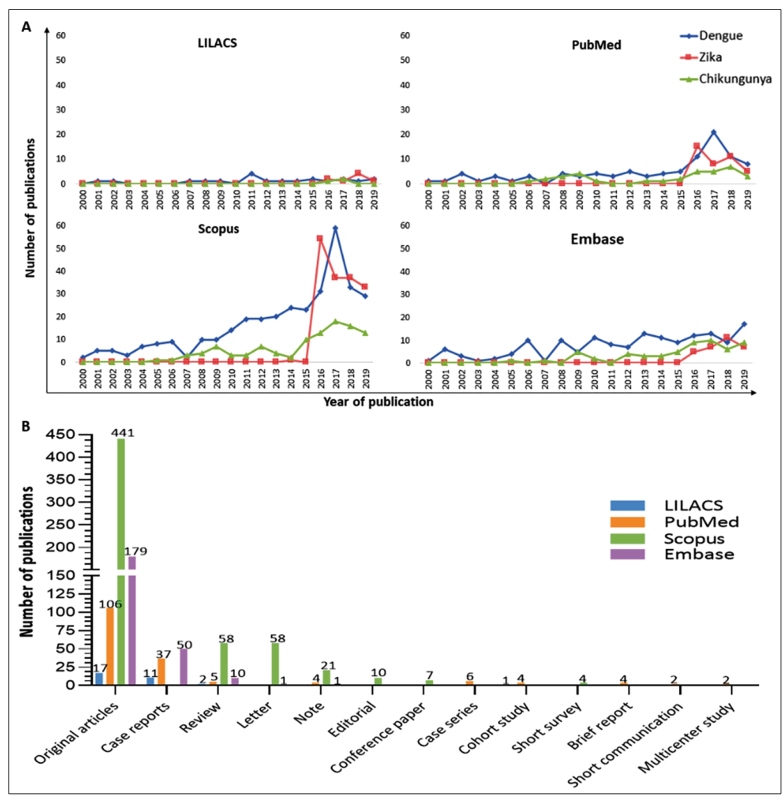
(A) Number of publications per year from 2000 to 2019 in LILACS, Pubmed, Scopus, and Embase databases retrieved using the isolated keywords “dengue”, “Zika”, “chikungunya”, and “CSF”. (B) Numbers of types of publications from 2000 to 2019 in the LILACS, Pubmed, Scopus, and Embase databases retrieved using the isolated keywords “dengue”, Zika”, “chikungunya”, and “CSF”.

There was a considerable change in the number of articles published between 2016 and 2019. In the LILACS database, 17 publications were identified: six pertaining to the keywords “dengue” and “CSF,” eight to “"Zika” and “"CSF”, and three to “chikungunya” and “CSF”. In the PubMed database, 110 publications were identified: 51 pertaining to the keywords “dengue” and “CSF”, 39 to “Zika” and “"CSF”, and 20 to “chikungunya” and “CSF”. In the Scopus database, 373 publications were identified: 152 pertaining to the keywords “dengue” and “CSF”, 161 to “Zika” and “CSF”, and 60 to “chikungunya” and “CSF”. In the Embase database, 115 publications were identified: 51 pertaining to the keywords “dengue” and “CSF”, 30 to “Zika” and “CSF”, and 34 to “chikungunya” and “CSF” (Figure 2A). 

#### Types of publications

Regarding the types of publications, 17 original articles, 11 case reports, two reviews, and one cohort study were retrieved from the LILACS database. Querying the PubMed database resulted in the identification of 106 original articles, 37 case reports, five reviews, six case series, four cohort studies, four brief reports, four notes, two short communications, and two multicenter studies. The Scopus database filtered 441 original articles, 58 reviews, 58 letters, 21 notes, 10 editorials, seven conference papers, and two short surveys (Figure 2B). Querying the Embase database resulted in the identification of 179 original articles, 50 case reports, ten reviews, one letter, and one note. Among the most common article types, there were 743 original articles, 98 case reports, and 75 reviews.

## DISCUSSION

The arboviruses DENV, ZIKV, and CHIKV are present in more than 120 subtropical and tropical countries. They emerge in poor urban areas and in the peripheral parts of the world. It is estimated that approximately 4 billion people are exposed to the risk of DENV, ZIKV, and CHIKV infections^19^. According to an epidemiological survey conducted by the Pan American Health Organization (PAHO) and the World Health Organization (WHO) in 2017, the epidemiological bulletin on DENV infection indicated a total of 579,645 reported cases, with 57,543 cases of ZIKV infections and 123,087 cases of CHIKV infections in the Americas^20^. In 2018, according to the epidemiological survey conducted by the PAHO and WHO, a total of 561,398 cases of DENV were reported, with 31,576 cases of ZIKV infections, and 94,239 cases of CHIKV infections in the Americas^21^. According to an epidemiological survey conducted by the PAHO and WHO in 2019, the epidemiological bulletin on DENV infection indicated a total of 3,140,872 reported cases, a total of 33,896 cases of ZIKV and 177,469 cases of CHIKV infections in the Americas^22^ *.*

This study reviewed the literature that describes the presence of DENV, ZIKV, and CHIKV in the CSF in different neurological conditions (encephalitis, congenital syndromes, microcephaly, meningitis, and GBS), as reported in various types of studies (case reports, original articles, and short communications). The differences in the number of searches accessible among the databases were justified by the information obtained as a result of each of the searches. LILACS contains only Latin American data. The PubMed database comprises the medical libraries of the United States and 70 countries. Scopus is the world’s largest database and covers more than 5,000 international publishers. The Embase database has international coverage and contains more than 32 million records from more than 8,500 journals. This characteristic reflects its greater ability to filter scientific papers.

In 2015, no studies were identified in any of the analyzed databases when the keywords “Zika” and “CSF” were combined. The incidence of GBS associated with ZIKV infection increased in the epidemics of 2015 and 2016 in Brazil, Colombia, the Dominican Republic, El Salvador, Honduras, Suriname, and Venezuela^23^ ^,^ ^24^ ^,^ ^25^ ^,^ ^26^ ^,^ ^27^ *.* At this time, an increase in the number of microcephaly cases was recognized in Brazil during the ZIKV epidemic. This finding highlights the possible relationship between the neurological condition and infection with the virus. Retrospective studies also found a proliferation in other congenital brain abnormalities during the ZIKV epidemic in 2013 in French Polynesia^24^ ^,^ ^25^. Therefore, the Centers for Disease Control and Prevention (CDC) confirmed this relationship based on epidemiological evidence. In February 2016, the WHO stated that the recent association of ZIKV infection with cases of microcephaly and other neurological disorders such as GBS constitutes a public health emergency of international interest, and made the notification of arboviruses such as ZIKV compulsory^28^. With this change, the growth of publications that involved these arboviruses was evident, and the co-circulation of these arboviruses became more notorious; the importance of the CSF for the neurological diagnosis of this condition was also recognized.

Brazil had the largest number of available scientific papers. Dengue fever, chikungunya fever, and acute disease caused by ZIKV are compulsory notifiable diseases in the country, and are included in the National List of Compulsory Notification of Diseases, Diseases, and Public Health Events, unified by Consolidation Ordinance No. 4 (September 28, 2017) from the Brazilian Ministry of Health. The data recorded by the Health Surveillance Secretariat demonstrate the epidemiological relevance of these arboviruses. In 2016, 2017, 2018, and 2019, the total reported probable case numbers related to DENV in various publications (1,487,924; 249,056; 247,393; 1,544,987), ZIKV (211,770; 17,338; 8,024; 10,768), and CHIKV (263,598; 185,605; 85,221; 132,205) infections in Brazil were significant^29^ ^,^ ^30^ ^,^ ^31^ ^,^ ^32^. We demonstrated that in the same period, there was an increase in the number of publications in the databases that related arboviruses with the use of CSF for diagnosis. It is important to highlight that despite the risks inherent to patients undergoing lumbar puncture, CSF analysis may be essential for the diagnosis of infections of the central nervous system (CNS), especially in tropical countries. Knowing this relevance, in Brazil, we have a culture of doing this type of analysis, and this is reflected in the greater number of Brazilian articles using CSF samples for the diagnosis of neuroarbovirosis.

It is estimated that 0.5-21% of patients with dengue develop neurological manifestations^33^. In CHIKV infections, 16% of patients are estimated to have neurological symptoms^34^. In ZIKV infections, it is estimated that 13.4-33.3% of patients may have neurological signs 15 to 30 days after infection^35^. In a previous study, our group found 31% of cases of neuroinvasive arboviruses (DENV/CHIKV) by RT-PCR and specific IgM in the CSF and/or serum in patients with suspected infectious or post-infectious neurological disorders (encephalitis, GBS, optic neuritis, neuromyelitis optic spectrum disorder, polyneuropathy, and myelitis). This analysis showed the importance of routine CSF investigations in endemic areas^36^.

The etiopathogenesis of neurological manifestations may occur through the direct action of the virus, by autoimmune mechanisms, hemorrhage, and the metabolic disorders it causes^6^. According to the literature, encephalitis is the most common neurological manifestation of DENV infections^17^, but it can also occur in ZIKV and CHIKV infections^11^. It since the 1960s, CHIKV infection has been known to affect CNS^37^ *.* In 2016, a cohort study was conducted at *La Réunion Island* reported CHIKV infections in 42% of encephalitis cases^38^. We highlight here the increase in the amount of research involving congenital syndrome cases and microcephaly related to ZIKV infection, CSF-confirmed arbovirus diagnoses, and disease-related complications after the occurrence of epidemics. Similarly, from 2000 to 2019, the main confirmed neurological diagnoses related to these arboviruses were encephalitis, congenital syndromes, and microcephaly. CSF analysis is very important to diagnose newborns with congenital syndrome/microcephaly due to infection by ZIKV. 

Our analysis showed that from 2000 to 2019, less than 1.1% of the scientific publications in the four databases used CSF analysis to investigate arboviruses (DENV, ZIKV, and CHIKV) in cases with neurological manifestations. As discussed above, considering the prevalence of neuroarboviruses and the importance of CSF for diagnosis, the number of scientific publications found in the area is underestimated. Therefore, neurological diseases caused by these arboviruses may be underdiagnosed. This is evident from our results which showed that only 8.03% of the suspected cases were diagnosed. Of these, 75% underwent CSF analysis alone or in association with other fluids. The detection of the specific IgM was the methodology that contributed the most to the diagnoses. Despite advances in the number of arbovirus investigations and the confirmation of CSF as an essential clinical sample in suspected cases with neurological manifestations, further research and more studies are needed. In addition, the authors do not exclude the possibility of a possible analysis bias in some of the studies included in this review. Among these biases, the sample size in the study, the statistical significance of the main result of a given study, and type of funding for some studies, may be included.

 The increase in recent years in the number of cases with a co-circulation of arboviruses (for example, DENV, ZIKV, and CHIKV) worldwide has led to a continuing need for research to clarify the pathophysiology of neurovascular diseases and complications caused by arboviruses. CSF analysis contributes to the diagnostic confirmation and elucidation of the neuropathogenesis of CNS tropical neurological diseases such as arbovirus infections. CSF analysis also plays a crucial role in excluding other possible causes of neurological disorders. In cases suspected of neurological manifestations due to viral infection, neuroarboviruses should be investigated in endemic areas. Despite the high morbidity and lethality of these arboviruses and their neurological complications, we emphasize that CSF is still rarely used for elucidation, diagnostic support, and research.

## Figures and Tables

**SUPPLEMENTARY FIGURE:**
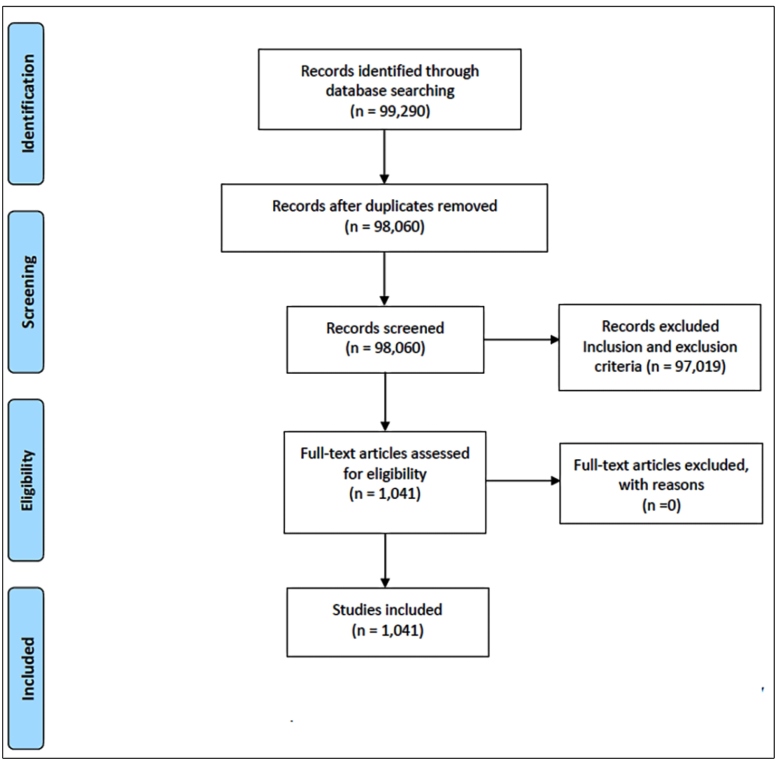
PRISMA chart displaying the article selection process from January 2000 to December 2019.

*From:* Moher D, Liberati A, Tetzlaff J, Altman DG, The PRISMA Group (2009). *P*referred *R*eporting *I*tems for *S*ystematic Reviews and *M*eta- *A*nalyses: The PRISMA Statement. PLoS Med 6(7): e1000097. doi: 10.1371/journal.pmed1000097 For more information, visit http://www.prisma-statement.org.
